# The Future of St. Bartholomew's Hospital

**Published:** 1903-11-14

**Authors:** 


### HOSPITAL ADMINISTRATION.

### CONSTRUCTION AND ECONOMICS.

### THE FUTURE OF ST. BARTHOLOMEW'S HOSPITAL.

AN APPEAL TO THE MEDICAL STAFF.

Mr. Andrew Motion, chairman of the Cannon Brewery, St. John Street, Clerkenwell, E.C., writes to us as follows :—

"The present position of the controversy regarding the provision of an adequate scheme for the reconstruction of St. Bartholomew's Hospital is well calculated to cause widespread anxiety and disquiet. The official proposals include an appeal to the public for a sum of £350,000—an appeal which obviously must fail unless the public judgment is first convinced. At the same time experts who speak with authority denounce the official scheme as seriously defective in respect both to present demands and future development. It is pointed out, for example, that the received plans include such a highly objectionable feature as the retention for the reception and treatment of in-patients of buildings which have been used for this purpose for at least 150 years, and in which it is impossible to obtain a proper supply of sunlight and fresh air. Further, the hygienic condition of the hospital will be made dangerous by adding an additional story to the north wing, and by crowding an already overcrowded site with many additional buildings. The scheme, in short, instead of representing the highest level of hospital construction and sanitary efficiency will merely put a modern gloss on old faults, and will provide a hospital having grave and essential defects. The corollary is inevitable, that the future St. Bartholomew's will be promptly recognised as an exhibition of the folly of half measures, and that its decline in public and professional estimation is certain. This, in essence, is the criticism passed by responsible voices on a plan now before the public for the reconstruction of a hospital the premier position of which, in relation both to historical associations and practical achievement, is recognised in all parts of the Empire. This fact alone is sufficient to show that the scheme is predoomed to failure. For efficiency the public sympathy may readily be enlisted; but it has no voice or help for a policy of shreds and patches.

"Criticism in the present instance, however, has not merely a destructive aspect. On the contrary, it propounds an alternative scheme which, if adopted, will secure for the hospital everything that modern experts advise, and will at the same time guarantee the opportunity for such developments as are probable in the future—the estimated outlay here being a sum of £600,000.

"These, in brief, are the rival policies presented to the public, and it needs no special gift of discernment to anticipate that the more radical proposal is the more likely to attract interest and support. St. Bartholomew's has a wide and well-deserved fame. Its traditions of efficiency and success may be traced through the medical history of 800 years. From its walls have issued for generations countless numbers who in all parts of the world have practised the art of healing. Its physicians and surgeons include the many names which are the cherished heritage of the profession. Enthusiasm on behalf of such an institution only waits to be aroused. There now exists an opportunity to add to the noble history of the past the developed efficiency which the modern spirit requires. Is it reasonable to believe that energy will awake on behalf of a proposal to meet this opportunity with half-hearted and temporising measures? Certainly it may be taken for granted that the controversies of the last 12 months have developed in the public a feeling of hesitation and doubt, and that the more recent criticisms command a degree of confidence which official silence will altogether fail to disturb. Before they offer their support the public will insist on knowing all the facts. In this respect one of the most remarkable features in this prolonged controversy has been the absence of any expression of opinion by the medical staff of the hospital. Doubtless the views of the staff were officially submitted to the Mansion House Committee, but they have never reached either the governors of the hospital or the general public. The responsibility of the staff in this matter is a considerable one. They are in a very special sense aware of the existing defects in the hospital. They are also aware of the changes necessary to remedy these defects. Further, it rests with them to estimate the probable future demands both of hospital treatment and medical education. Their judgment and decision on these points must necessarily carry substantial weight, and in the essential interests of the hospital it is imperative that their opinions be publicly pronounced. With them in a large measure rests the decision whether 'Bart's' shall continue and enlarge its noble history, or shall dwindle into a second or third-rate position among the medical institutions of the metropolis. Public opinion will certainly demand that London shall have at least one modern hospital and medical school replete in every respect. The inclination of the City and indeed of all parts of the country is to support the position and claim of St. Bartholomew's. But there are other candidates, and their virtues will not be neglected on merely sentimental considerations. It is only an efficient programme which will secure public support. There are rumours afloat that the silence of the hospital staff is due to a fear of offending one or more august patrons of the hospital. Considering that the most distinguished personage in the realm and his Royal son have both identified themselves with a policy of hospital thoroughness and efficiency, these rumours may be neglected. In any event the claim for a clear statement is imposed by the sanction of duty. It rests with the staff to satisfy public opinion, and in the absence of this, the financial success of any scheme is more than doubtful. They have still another opportunity. 'Bart's' men, proud of their *alma mater*, are to be found in influential positions all over the world. A courageous appeal from the present staff, accompanied by the assurance that the future of the hospital and school are at stake, would go a long way to raise the money necessary for a complete reconstruction. The precedent of 'Guy's' is a full justification for this statement. But so long as the medical staff stands silent so long will there be doubt and mistrust, and the public will remain aloof. The opportunity for a large and worthy success now exists. Is it to be lost through a faint hearted and short-sighted policy, backed by the supineness of the medical staff and old Bart's men all over the country? I cannot believe it!

"I am organising a definite, purposeful, and sustained opposition to the Mansion House Committee's scheme, which, if persisted in, must ultimately ruin St. Bartholomew's Hospital. I am a Governor of St. Bartholomew's Hospital, and although the fight will be earnestly maintained by us laymen, the continued silence of the medical staff must render a difficult task doubly difficult. If it be true that the Medical Council made representations to the treasurer before the meeting of the Governors on the 5th inst. to the effect that the site must be larger and that the wards for the reception of patients must be entirely modern and up to date, that report has been kept back from the Governors. We ought, however, to know the facts."

### THE QUESTION OF RECONSTRUCTION.

### OPPOSITION TO THE MANSION HOUSE SCHEME!

A GENERAL Court of Governors of St. Bartholomew's Hospital was held on November 5th, Sir Trevor Lawrence the treasurer, presiding, to consider the report of the Mansion House Committee, and to decide what steps shall be taken in furtherance of the proposed appeal for funds. Of the 250 Governors, about 50 were present at the meeting. The following resolutions were handed to each Governor, and were moved *en bloc* by Mr. Cripps, K.C., M.P.

1. That this court desires to record its unqualified satisfaction that the Mansion House committee, after impartial and exhaustive inquiry, have come to the following conclusions, viz.:—

(*a*) That it is impossible, in the public interest, to remove the hospital from its present site;

(*b*) That the additions to and rearrangement and improvement of the existing buildings, as proposed by the Governors, are absolutely necessary for continued efficiency in the treatment of patients and for bringing the hospital in all departments up to the standard of modern sanitary and scientific requirements;

(*c*) That an appeal to the public to supply the cost of the requisite new buildings and improvements is fully justified, it having been shown to be impossible for the hospital to provide the same out of its own resources:

(*d*) That the reputation and character of the hospital have been fully vindicated, and that its administration has been conducted in a wise and enlightened spirit, with due regard to economy and to the best interests of the patients.

2. That the court begs to offer to the gentlemen who served on the Mansion House committee its cordial thanks for the large amount of time and the great attention bestowed by them on all matters connected with their inquiry

3 That, in the general scheme of rebuilding, provision be made for:—(*a*) additional operating theatres; (*b*) new casualty and out-patient department; (*c*) new nurses' home; (*d*) new quarters for the resident medical and surgical staff; (*e*) an isolation block and other new ward blocks to supply the place of those to be demolished; (*f*) new mortuary, post-mortem rooms, and pathological department; (*g*) internal structural rearrangement of the east, west, and south wings of the hospital.

4. That the house committee, with power to add to their number, be requested to determine, after consultation with the medical staff, by which of the several plans submitted by the architects, subject to such modifications as from time to time appear desirable, provision for the foregoing purposes can best be secured; but that it be an instruction to the house committee to carry out the recommendation of the Mansion House Committee as regards the out-patient department as shown on plan No. 6 laid before that committee.

5. That the first block to be built be the new casualty and out-patient departments, dispensary, etc, and the new operating-theatres; that the nurses' home be next built; and that the remaining blocks be built in such order as, having regard to their relative urgency, may be considered most expedient and convenient for the general work of the hospital.

6. That the medical staff be consulted from time to time on all details that affect the sanitary arrangements, the treatment of the patients, or the interests of the medical school.

7. That the treasurer and almoners be authorised to make arrangements from time to time for the temporary use of such of the Christ's Hospital buildings as may be necessary for carrying on the work of the hospital. Also, that they be given authority to make any provision they may think proper for officers and servants whose present quarters are destroyed or interfered with thereby.

8. That application be made to Parliament in the ensuing session for power to the Governors to use, for the purposes of the hospital in connection with the proposed new buildings and improvements, the site of the church of St. Bartholomew-the-Less and the burial ground adjoining within the hospital precincts, and for the union, for ecclesiastical purposes, of the parish of St. Bartholomew-the-Less with the adjacent parish of St. Bartholomew-the-Great.

9. That, after the Lord Mayor-elect has entered upon office, the treasurer and almoners do wait upon him with a request from this general Court of Governors that, at some convenient date, he will convene a meeting of the citizens for the purpose of raising the requisite funds for the new buildings and improvements needed.

Referring to the suggested alternatives that had been put forward, Mr. Cripps thought that as the Mansion House Committee had approved a plan for reconstructing the hospital, it was better to agree to it, and to proceed with the work at once rather than indefinitely to postpone the alterations with a view to the elaboration of a larger scheme embodying a further purchase of land. He urged that it was desirable the resolutions should be adopted unanimously, because otherwise the public would hesitate to subscribe the £350,000 needed.

Sir William Church seconded the motion.

Mr. Andrew Motion (Chairman of the Cannon Brewery) proposed as an amendment:—

That the best interests of this hospital require the purchase of more land from Christ's Hospital to a sufficient extent to enable modern wards for 700 beds to be built and the whole institution to be remodelled and brought up to date.

He objected to the Mansion House Committee's proposal to commit the hospital to an expenditure of £350,000, because it meant the retention of the existing wards as the main accommodation provided for the treatment of patients. Such a scheme must ultimately be fatal to the best interests of St. Bartholomew's and its medical school. Should it be persisted in, he and the influential friends associated with him would feel bound to organise opposition to the raising of any money to carry out the scheme, and so save the hospital from the evil fate with which it was threatened. The great bulk of the Governors were absent, and those there had only had the resolutions placed in their hands on their entrance to the Court-room. Speaking as a representative of the business interests of the City of London, he declared that at least two acres in addition should be purchased, and that a new and modern hospital should be erected, on some such plan as that prepared by Sir Henry Burdett, worthy of the City and of that great institution. In his view the meeting should be adjourned. Until the Governors had had an opportunity of reading the full evidence given before the committee by Sir Henry Burdett, the Medical Council, and other witnesses, of which hitherto they had only received a very imperfect summary, they were not in a position to express a final opinion on the important issues underlying the present crisis. The Medical Council had reported that the present site was inadequate, and that the hospital should be rebuilt. For these reasons, while he and his friends, and he believed the majority of the thinking public who support hospitals in this country, were prepared to generously support an adequate scheme, worthy of the traditions of so great an institution, they were determined to use their utmost endeavours to prevent the tinkering and injurious proposals embodied in the Mansion House scheme from being imposed on the hospital, to its permanent injury. He, therefore, urged the Governors present to agree to adjourn the consideration of the matter in the light of the evidence which should be sent to them, when he had no doubt the result would be the adoption of an adequate scheme, and the raising of all the money necessary. Failing that, he wished it to be clearly understood that neither he nor his friends would contribute anything to the £350,000 scheme, on account of its temporising character. They were desirous of uniting the Governors so as to secure the full support of the public, and in the absence of such unanimity the appeal must fail, and deserved to fail. He urged an adjournment for a month, to allow them to send a copy of the full evidence to each of the Governors, and the holding of a conference with the object of securing united action in the interests of St. Bartholomew's Hospital, which all must have at heart.

On being put to the meeting the resolutions were adopted, after which there was some discussion on them in detail. Referring to the last paragraph of the Mansion House Committee's conclusions, that the reputation of the hospital had been vindicated, and that it had been conducted to the best interests of the patients, Mr. Butlin, one of the surgeons, said the out-patient department was in a disgusting state, unfit for the treatment of human beings, and Mr. Howard Marsh, another member of the staff, declared that its condition was nothing short of a scandal.

On the motion of Sir Wm. Church, it was decided to add words giving power to the House Committee to add to its number, and providing that nothing should be done until the plans had been settled in consultation with the medical staff.

